# Concordance between Body Composition Indices Measured with Dual-Energy X-Ray Absorptiometry and Bioelectrical Impedance Analysis in Obese Children in Sri Lanka

**DOI:** 10.1155/2021/6638057

**Published:** 2021-02-12

**Authors:** Mawanane Hewa Aruna Devapriya de Silva, Ruwani Punyakanthi Hewawasam, Sarath Lekamwasam

**Affiliations:** ^1^Department of Paediatrics, Faculty of Medicine, University of Ruhuna, Galle, Sri Lanka; ^2^Department of Biochemistry, Faculty of Medicine, University of Ruhuna, Galle, Sri Lanka; ^3^Department of Medicine, Faculty of Medicine, University of Ruhuna, Galle, Sri Lanka

## Abstract

Dual-energy X-ray absorptiometry (DXA) is the reference standard in the measurement of body composition indices. But, its utility is limited due to the high cost, expertise required, lack of portability, and restricted availability. Therefore, bioelectrical impedance analysis (BIA) has gained recognition in resource-limited settings for the measurement of body composition indices in the screening of children for childhood obesity. To determine whether BIA represents a viable alternative to DXA in the assessment of body composition in obese children in the community setting in Sri Lanka, the concordance between BIA and DXA was determined. Fat mass (FM), percentage fat mass (%FM), and fat-free mass (FFM) were measured in 97 obese children using DXA and BIA, and the concordance between the methods was analyzed using independent sample *t*-test, regression analysis, and Bland-Altman plots. Significant mean differences were observed between DXA and BIA in measuring FM and FFM. However, high correlations were seen in DXA- and BMI-derived FM and FFM measurements (FM *r* = 0.92 and FFM 0.83, *P* < 0.001 for both). Compared to DXA, BIA overestimated FM and %FM and underestimated FFM. When compared with DXA-derived measurements, the accuracy errors (SEE) of BIA for FM, FFM, and %FM were relatively higher in boys (3.56 kg, 4.49 kg, and 5.46%, respectively) than in girls (2.44 kg, 3.72 kg, and 3.5%), respectively. BA plots showed a systematic error in the measurements of FM, FFM, and %FM in both sexes. Despite the limitations inherited, BIA is a viable alternative to DXA for the measurement of body composition in obese children of 5-15 yrs. The accuracy errors observed, however, need to be taken into consideration when interpreting results at the individual level.

## 1. Introduction

Overweight and obesity in childhood are serious public health concerns, both in developed and developing countries. According to the World Health Organization (WHO), the prevalence of overweight or obesity between the ages 0 and 5 years increased from 32 million in 1990 to 41 million in 2016, globally. Furthermore, the majority of overweight or obese children were reported from developing countries where the rate of increase has been more than 30% higher than that of developed countries. It has been estimated that 10% of schoolchildren worldwide are overweight and a quarter of this group are obese [1]. An analysis in 2014 involving 836 school children (431, 51.6% girls) in the Galle municipal area in Southern Sri Lanka showed the prevalence of overweight or obesity to be 10.3% [2].

Obesity, which is characterized by the accumulation of excess body fat, is a major risk factor of metabolic and cardiovascular disease, such as type 2 diabetes mellitus, nonalcoholic fatty liver disease, and dyslipidemia [3]. Apart from body fat content, percentage fat mass (%FM) has also been identified as a risk factor of metabolic syndrome [4]. Body mass index (BMI) is widely used as a surrogate measure of obesity due to its simplicity, general applicability, and the accuracy of the basic measurement [5]. Several studies in the recent past, however, have shown that in clinical practice, the criteria for overweight and obesity that are based only on the 85^th^ or 95^th^ percentile of BMI may not accurately identify children who are at a greater health risk [6]. Further, BMI and waist circumference, which are commonly used to assess central obesity, do not distinguish between fat mass (FM) and fat-free mass (FFM) [7]. The WHO, recognizing the inaccuracy of a universal cut-off point to define obesity, has suggested that body composition studies are required in different populations to study the relationships between body fat content, BMI, and body size to formulate ethnicity-specific BMI thresholds. Asians have higher body fat content than Caucasians and also they differ with regard to the associations between BMI, %FM, and health risks compared to European populations [8]. Therefore, an accurate assessment of FM and FFM in obese children is important to assess the interrelationship between body compartments and their behavior with therapeutic interventions. A key objective of the management of obesity is to reduce FM while preserving FFM during weight loss [9].

Currently, many options are available to measure body fat mass, and these include relatively simple field techniques, such as bioelectrical impedance analysis (BIA) and skinfold-thickness measurements, as well as more sophisticated techniques such as dual-energy X-ray absorptiometry (DXA) and quantitative computed tomography (CT) and magnetic resonance imaging (MRI) [10].

BIA has many advantages such as portability, simplicity, noninvasiveness, speed of measurements, safety, and affordability. Since minimal subject corporation is required, it is ideal for routine practice in children [11]. The principle of BIA measurements is based on the resistance to the flow of an electrical current passing through the body. By measuring the impedance to the flow of a small current, total body water (TBW) is estimated and assuming that TBW constitutes a fixed percentage of lean mass, body composition is estimated. Specific equations have been developed to predict body composition using height, weight, and impedance of an individual [12]. Previous studies, however, have reported that the validity of BIA may be limited in extremely obese subjects due to their altered body geometry and body water distribution. Also, the accuracy of BIA has not been established in Asian children especially in those with obesity or excess of adiposity [1].

DXA is considered the reference standard for the measurement of body composition, FM, lean tissue mass, and bone mineral content and also regional fat distribution. The assessment of body composition by DXA involves minimal radiation exposure. The utility of DXA, however, is limited due to the high cost, expertise required to acquiring and analyzing scans, lack of portability, restricted availability, and the weight limit. These criteria often limit the availability of DXA in most clinical settings [13].

Since we were unable to find local studies examining whether BIA represents a viable alternative to DXA for the assessment of body composition in overweight and obese children in clinical settings, the present study was designed to compare the level of agreement between BIA and DXA in the assessment of FM, %FM, and FFM in obese children in Southern Sri Lanka. In these analyses, DXA-derived measurements were considered as the reference standard.

## 2. Patients and Methods

### 2.1. Subjects

The study protocol was approved by the Ethical Review Committee of the Faculty of Medicine, University of Ruhuna, Sri Lanka (Ref: 09.03.2016:3.10). Ninety-seven children (57 boys) aged 5-15 years who had a BMI ≥ 85^th^ percentile for age and gender based on the CDC 2000 growth charts were recruited to the study from the field study area of the Faculty of Medicine, Galle. Parents were informed about the purpose of the study and the procedures involved, and written consent was obtained. Children attended the laboratory after an overnight fast and were asked to refrain from participating in strenuous exercise prior to the research to avoid perturbation of the hydration status which can interfere with the BIA measurement. They were examined by the principal investigator (MHADdS) prior to the commencement of the study and were confirmed as healthy. Children with dysmorphic syndromes and those who were obese due to iatrogenic causes were excluded from the study.

### 2.2. Measurement of Height and Weight

Height was measured without shoes with a wall-mounted stadiometer (Seca, Birmingham, UK) to the last completed 0.1 cm after keeping heel, buttocks, back of shoulder, and occiput in the vertical plane and head in the horizontal Frankfurt plane. The weight was measured with minimal light indoor clothing, to the closest 0.1 kg using a calibrated electronic weighing scale (Nagata, BW-110H CAP, Taiwan). BMI was calculated as weight (kg) divided by height (m) squared.

### 2.3. Measurements Made by Dual-Energy X-Ray Absorptiometry and Bioelectrical Impedance Analysis

DXA and BIA measurements were obtained on the same day. Subjects, after overnight fasting, underwent BIA first followed by DXA evaluation with a two-hour gap.

Body FM and FFM (lean mass and bone mineral content) were measured with a DXA scanner (Hologic Discovery W, Hologic Inc., MA, USA, Version 4.6.0.2 application software) adhering to the manufacturer's recommendations. Measurement were taken in light clothing without any metal items in their clothing or elsewhere while the study subjects were lying in a supine position. Scans were analyzed by inbuilt software (for obese subjects) provided by the manufacturers. In vitro precision of the machine was checked on each scanning day with whole body phantom provided by the manufacturer.

A whole body impedance was measured by a foot to foot BIA analyzer (Tanita SC-240A, Tanita Corporation, Tokyo, Japan). Subjects were asked to stand on the metal sole plates on the machine, and gender and height details were entered to the system. The body FM and lean mass were calculated using the built-in prediction software.

### 2.4. Statistical Analysis

Statistical analyses were performed for the entire sample and for boys and girls separately using IBM SPSS statistics, version 25. Independent *t*-test was used to compare subject characteristics. Linear regression analyses were used to determine the relationships between DXA and BIA for FM, %FM, and FFM. The standard error of estimate (SEE) was used to determine the degree of accuracy error of BIA compared to DXA. Bland-Altman (BA) pairwise comparisons were used to assess the agreement between the same type measurements generated by DXA and BIA. In BA plots, the limits of agreement were defined as mean difference ± 1.96 SD. *P* was adjusted by the Bonferroni method for multiple comparisons.

## 3. Results

**Figure 1:**
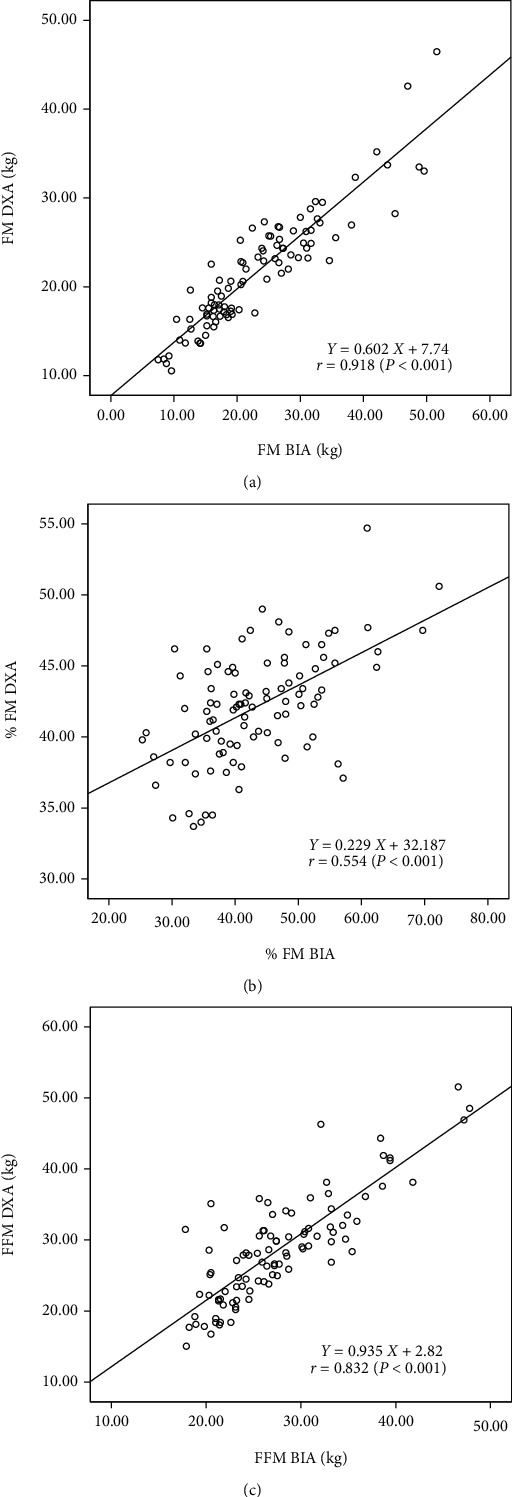
Regression plots between (a) fat mass (FM), (b) percentage fat mass (%FM), and (c) fat-free mass (FFM) assessed by DXA and BIA.

**Figure 2:**
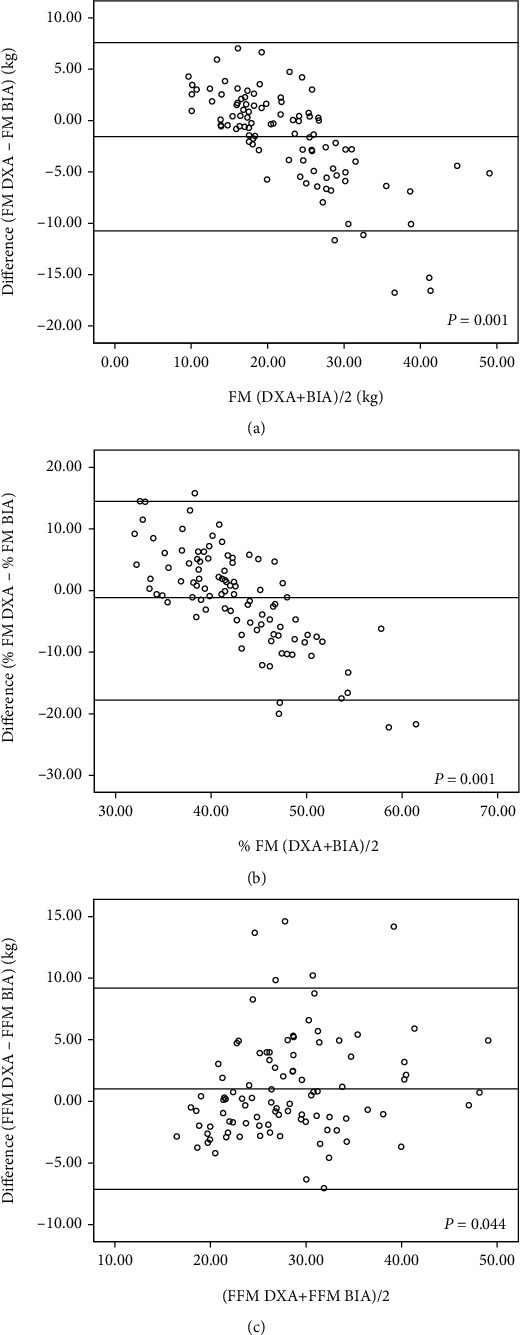
Bland-Altman plots comparing the accuracy of BIA in measuring fat mass (a), percentage fat mass (b), and fat-free mass (c) The middle solid line represents the mean difference between DXA and BIA, and the upper and lower lines represent 95% limits of agreement.

**Figure 3:**
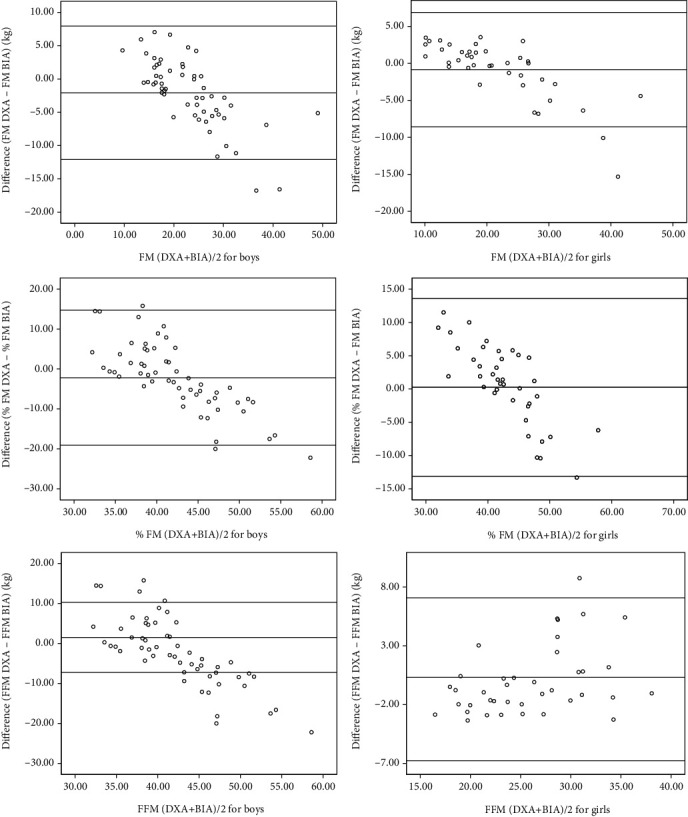
Bland-Altman plots comparing the accuracy of fat mass (FM), percentage fat mass (%FM), and fat-free mass (FFM) measurements by DXA and BIA in boys and girls. The middle line represents the mean difference between DXA and BIA, and the upper and lower lines represent 95% limits of agreement.

**Figure 4:**
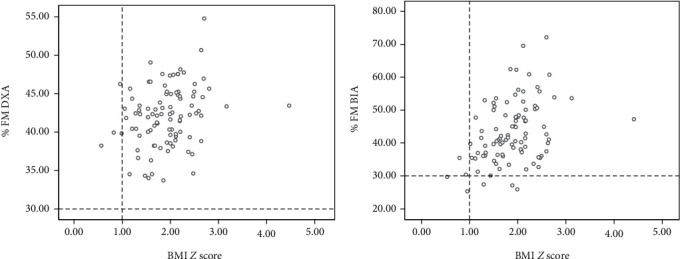
Differences between DXA and BIA in the measurement of percentage fat mass in overweight or obese children.

In the study sample, boys and girls were not different with regard to age, weight, height, BMI, and body composition indices except for the FM by BIA. Compared to DXA, BIA overestimated FM and %FM and underestimated the FFM. Further, the mean differences of body composition indices made by DXA and BIA were wider among boys compared to girls (Table 1).  Table 2 shows the mean differences and 95% CIs between measurements made by DXA and BIA. Significant differences were seen in the FM (*P* = 0.001) and FFM (*P* = 0.018) measurements made by DXA and BIA indicating an accuracy error with BIA. When mean differences were reanalyzed according to gender, the differences were narrower and not significant among girls while they were wider and significant among boys indicating that the accuracy error is mainly confined to boys (Table 3). This is further highlighted by higher SEE seen among boys compared to girls (Table 3). In the regression analysis, %FM estimated by two methods showed a higher scatter compared to FM and FFM (Figure 1). This was further confirmed by higher SEE observed for %FM when compared with FM and FFM (Table 3). Although a concordance of the measurements made by the two methods was seen in the BA plot analysis (95% values were within the limits of agreement, Figures 2 and 3), values showed lack of randomness and drifting, and this was particularly seen among boys compared to girls. Furthermore, %FMs measured by both DXA and BIA showed a linear relationship with BMI (Figure 4).

## 4. Discussion

This study compared body composition data of children aged 5-15 years with obesity in Southern Sri Lanka measured by two different body composition methods. DXA, which is considered a reference standard in body composition measurement in adults, is not considered a feasible method in pediatric patients as the procedure takes up to twenty minutes, and it requires the patient to stand still during the procedure. Although the radiation exposure in a single DXA measurement is as low as 0.001 mSv, much less than a standard chest or dental radiograph [14], parents are concerned about the radiation risk in DXA. Moreover, the necessity of trained radiology personnel, cost of the device, and restricted availability are the other limitations of DXA technology. The feasibility, safety, and portability make BIA the frequently used method of body composition analysis, especially in children [15]. BIA measurements are based on body water content; hence, the accuracy of BIA is likely to vary according to age and body size. Most of the studies on BIA performance have been done in European countries, and there is a scarcity of studies done in South Asian countries.

In the present study, compared to DXA, BIA overestimated FM and %FM and underestimated FFM, and these errors were particularly seen among boys (FM by 3.56 kg, %FM by 5.46%, and FFM by 4.49 kg) than girls (FM by 2.44 kg, %FM by 3.72%, and FFM by 3.5 kg). FM and FFM measured by BIA were significantly different from values obtained by DXA. Although more than 95% of data points were within the limits of agreement in BA plots, a systematic error was seen, particularly in FM and %FM where a downward drifting of differences between the two methods was seen as FM increased. This indicates a higher measurement error of BIA in those with higher FM or %FM, and this error was seen in both sexes.

A statistical analysis taken singularly is unable to ensure the measurement accuracy of a technique, and the final conclusion is made based on multiple statistical test results. Ideally, the mean difference between the two sets of data should be nonsignificant, and the value should be close to zero. Further, narrow SD of the mean difference would indicate less scatter of the measurements and higher precision of the estimate. While SEE would indicate the degree of accuracy error, BA plots would reflect the concordance between the two sets of data in the entire range of values. Based on our results, it can be concluded that BIA would be an acceptable method to estimate body composition of obese children in this age group. Further, the accuracy errors in different measurements need to be considered in interpreting results. Studies have shown that BIA overestimates [16] as well as underestimates %FM and FM^17^. We believe that technology-related factors and variations in characteristics of study samples could partly explain this inconsistency.

The mechanisms responsible for the overestimation of %FM and FM by BIA observed in our study are unclear. However, it is justifiable to hypothesize that the discrepancy could partially be due to the characteristics of the subjects we studied. Body composition analyses in BIA are built on calculations that are based on the electrical impedance or the resistance to the flow of electrical current through body tissues. This information is applied to gender-specific equations to calculate either body density or fat-free mass. Body density is used to calculate %FM according to a standard densitometric formulae based on the assumption that the densities of fat and FFM are constant [17]. In case of FFM, it is directly used to calculate %FM with body weight. Therefore, BIA assumes that the density of each tissue is constant between individuals, and with time [18]. Since BIA seems well suited for daily or frequent body composition assessment in children, establishment of specific equations for the accurate assessment of body composition in children using BIA is timely. It was also reported that BIA measurements may be affected by meals, physical activity, and other variables that change the participant's hydration state [19]. There are also age-related changes in body composition during the adiposity rebound. Recent studies have considered the associations between selected lifestyle and environmental factors such as physical activity, diet, television viewing, breastfeeding, and body composition during the adiposity rebound [20, 21]. Due to the fact that BIA estimates body composition considering two compartmental models where FFM is measured directly, higher measurement accuracy and correlation can be expected for FFM than FM. Studies, however, have shown higher correlation coefficient for FM than FFM measured by BIA and DXA. In a similar analysis, Achamrah et al. observed *r* = 0.89 and *r* = 0.95 for FFM and FM, respectively, and these values are concordant with our observations [22].

Studies have previously explored the effect of ethnicity on the measurement of body composition of different ethnic groups. A study comparing the total body water, height, weight, age, and gender of 125 Caucasians and 89 African-Americans revealed that the prediction equations for total body water differ between the two racial groups [23]. Another study which assessed the association between ethnicity, body mass index, and bioelectrical impedance in study subjects from 10 different ethnicities also concluded that ethnicity-specific references are needed when applying BIA [24].

Body geometry also contributes to the differences in BIA and DXA values. Body extremities that are relatively long with a small diameter give rise to higher impedance values, but the trunk contributes minimally to total body impedance due to the presence of about one-half the conductive mass [25]. In obese individuals, a higher proportion of water is located in the trunk, which would lower total body impedance, resulting in an underestimation of FFM. It has been assumed according to previous reports that the total body water of lean body mass is 73.2%, but it changes during obesity [26]. Therefore, effects of hydration status and body geometry has a direct effect on the systematic bias observed with BIA measurements, which may lead to either over- or underestimation of FFM and FM with BIA [25].

However, the total body water could be higher in children [27] particularly in those with obesity [28], which could cause an overestimation of the lean body mass and, consequently, an underestimation of BF% measured by BIA. Similarly, extracellular water is higher in obese adults and children [29], which could also lead to an underestimated value for the BF%. Moreover, differences reported between DXA and BIA methods to estimate %FM might be due to the fact that BIA devices rely on the two-component (2-C) model, whereas DXA uses a three-component (3-C) model. Thus, the 2-C model is directly affected by water and electrolytes, whereas the 3-C model is not [30].

Only a few studies were reported from Asia on the comparison of body composition between DXA and BIA. A study conducted in Korea reported that BIA overestimated the total body fat by 2.54 kg, and significant changes in %FM were observed compared to DXA (*P* < 0.001) [31]. Another study conducted in Hong Kong reported that BIA overestimated FM by a mean of 1.93 kg with a much narrower limit of agreement [19]. We, however, feel that further studies are needed in Sri Lanka with a larger sample size and wider BMI and age ranges to obtain more accurate information and better understanding of the differences between BIA and DXA. Therefore, our data can be used as a platform for future studies on this subject.

The study has several limitations including the small sample size and use of a BIA device which was not programmed for children and obese subjects. We, however, used DXA as the reference standard, and this can be seen as a strength of the study. Also, all the measurements were made under the direct supervision of investigators by trained technical staff adhering to the standard protocols. In order to avoid between assessor variations, the same technician was involved in the measurement of a particular measurement.

## 5. Conclusion

There were significant differences in DXA- and BIA-derived FM, %FM, and FFM in obese children of 5-15 years. The accuracy errors were higher among boys compared to girls. The results indicate some limitations of BIA in measuring body composition of obese children. Clinicians need to be fully aware of the limitations of BIA as an alternative in the estimation of body composition of overweight and obese children, until unrestricted access to DXA is available in Sri Lanka.

## Figures and Tables

**Table 1:** Descriptive data of 97 study participants.

Variable	Boys (57)		Girls (40)		All (97)		*P* value
	Mean	SD	Mean	SD	Mean	SD	
Age (years)	10.8	2.4	10.3	2.6	10.6	2.5	0.35
Height (cm)	143	11.8	142	13.6	143	12.5	0.54
Weight (kg)	52.0	13.7	49.6	13.8	51.0	12.8	0.61
BMI (kg/m^2^)	25.7	3.4	25.2	4.1	25.5	3.7	0.53
FM DXA (kg)	21.8	6.1	22.1	7.1	21.9	6.5	0.81
FM BIA (kg)	24.4	9.6	21.9	10.1	23.4	9.8	0.022^∗^^#^
%FM DXA	41.4	3.6	43.2	4.2	42.1	3.9	0.21
%FM BIA	44.6	9.5	41.3	9.1	43.3	9.4	0.24
FFM DXA (kg)	29.5	7.4	27.5	7.5	28.7	7.5	0.09
FFM BIA (kg)	27.6	6.7	27.7	6.7	27.6	6.6	0.88

Abbreviations: BMI: body mass index; FM DXA: fat mass, dual-energy X-ray absorptiometry; FM BIA: fat mass, bioelectrical impedance; %FM DXA: percentage fat mass, dual-energy X-ray absorptiometry; %FM BIA: percentage fat mass, bioelectrical impedance; FFM DXA: fat-free mass, dual-energy X-ray absorptiometry; FFM BIA: fat-free mass, bioelectrical impedance. ^∗^*P* contrasts gender differences.

**Table 2:** Mean (SD) difference and 95% CI between the measurements made by DXA and BIA.

	Mean difference (SD)	95% confidence interval		*P* value
		Lower	Upper	
Fat mass (kg)	-1.57 (4.68)	-2.5134	-0.6184	0.001
Percentage fat mass	-1.16 (7.97)	-2.7778	0.4528	0.16
Fat-free mass (kg)	1.02 (4.17)	0.1760	1.8650	0.018

**Table 3:** Mean (SD) difference and 95% CI between the measurements made by DXA and BIA according to gender.

Variable measured	Mean difference (SD)	SEE	95% confidence interval of the difference		*P* value
			Lower	Upper	
Fat mass: boys	-2.08 (5.11)	3.56	-3.44	-0.71	0.004^∗^
Fat mass: girls	-0.85 (3.94)	2.44	-2.11	0.41	0.18
% fat mass: boys	-2.18 (8.62)	5.46	-4.49	0.13	0.06
% fat mass: girls	0.26 (6.82)	3.72	-1.92	2.44	0.81
Fat-free mass: boys	1.52 (4.48)	4.49	0.32	2.72	0.014^∗^
Fat-free mass: girls	0.32 (3.62)	3.50	-0.84	1.48	0.58

## Data Availability

The datasets used and/or analyzed during the current study are available from the corresponding author on reasonable request.

## Abbreviations

DXA: Dual-energy X-ray absorptiometry
BIA: Bioelectrical impedance analysis
FM: Fat mass
%FM: Percentage fat mass
FFM: Fat-free mass
BMI: Body mass index.
